# Promoting the Insertion of Molecular Hydrogen in Tetrahydrofuran Hydrate With the Help of Acidic Additives

**DOI:** 10.3389/fchem.2020.550862

**Published:** 2020-10-14

**Authors:** The Thuong Nguyen, Claire Pétuya, David Talaga, Arnaud Desmedt

**Affiliations:** ^1^Groupe Spectroscopie Moléculaire, ISM, UMR5255 CNRS—University, Bordeaux, France; ^2^Jet Propulsion Laboratory, California Institute of Technology, Passadena, CA, United States

**Keywords:** strong acid, hydrogen storage, tetrahydrofuran, hydrates, clathrates, Raman spectroscopy

## Abstract

Among hydrogen storage materials, hydrogen hydrates have received a particular attention over the last decades. The pure hydrogen hydrate is generated only at extremely high-pressure (few thousands of bars) and the formation conditions are known to be softened by co-including guest molecules such as tetrahydrofuran (THF). Since this discovery, there have been considerable efforts to optimize the storage capacities in hydrates through the variability of the formation condition, of the cage occupancy, of the chemical composition or of the hydrate structure (ranging from clathrate to semi-clathrate). In addition to this issue, the hydrogen insertion mechanism plays also a crucial role not only at a fundamental level, but also in view of potential applications. This paper aims at studying the molecular hydrogen diffusion in the THF hydrate by *in-situ* confocal Raman microspectroscopy and imaging, and at investigating the impact of strong acid onto this diffusive process. This study represents the first report to shed light on hydrogen diffusion in acidic THF-H_2_ hydrate. Integrating the present result with those from previous experimental investigations, it is shown that the hydrogen insertion in the THF hydrate is optimum for a pressure of *ca*. 55 bar at 270 K. Moreover, the co-inclusion of perchloric acid (with concentration as low as 1 acidic molecules per 136 water molecules) lead to promote the molecular hydrogen insertion within the hydrate structure. The hydrogen diffusion coefficient—measured at 270 K and 200 bar—is improved by a factor of 2 thanks to the acidic additive.

## Introduction

Hydrogen is the most abundant element on Earth and is considered as a clean and potential energy vector in the future. H_2_ storage and transportation are the subject of numerous studies. Among gas storage materials, hydrogen clathrate hydrates (also called hydrates) have received a particular attention over the last decades (Florusse et al., 2004; Veluswamy et al., 2014). Gas hydrates are crystalline inclusion compounds of hydrogen bonded water molecules forming cages encapsulating guest molecules (Sloan and Koh, 2008; Broseta et al., 2017; Ruffine et al., 2018). The pure H_2_ clathrate hydrate is generated only at extremely high-pressure (few thousands of bars) and at low temperature (~240 K) (Dyadin et al., 1999; Mao et al., 2002). The hydrate is then formed with H_2_ molecular species located in the small cages (SC) and in the large cages (LC) of the so-called sII hydrate (consisting of 16 SCs and 8 LCs) (Mao et al., 2002). To enable the storage of H_2_ under softer conditions (typically 50 bars and 280 K), the method consists in co-including H_2_ molecules with a second guest such as tetrahydrofuran (THF), to form a mixed THF-H_2_ sII hydrate (Florusse et al., 2004; Lee et al., 2005). However, THF molecules occupy the LC and H_2_ molecules are only in the SC, which leads to low hydrogen storage (<2 wt%) (Strobel et al., 2006; Mulder et al., 2008) and limits the potential applications (Nakayama et al., 2010). Since this discovery, there have been considerable efforts to optimize the storage capacities of H_2_ in clathrate hydrates through the variability of the formation condition, of the cage occupancy, of the chemical composition (by changing the promoter and its concentration) or of the cage structure (from clathrate to semi-clathrate) (Veluswamy et al., 2014).

Beyond the problematic of H_2_ storage capacity for potential applications, the H_2_ insertion mechanism plays is of fundamental interest. When H_2_ gas pressure is applied onto a powdered THF hydrate, the formation mechanism involves two steps: hydrogen adsorption onto the clathrate particle surface, followed by subsequent diffusion of hydrogen into the clathrate hydrate particle. The inter-cage diffusion represents the limiting step (timescale of the order of days), involving high activation energy (78.7 kJ/mol) (Nagai et al., 2008), confirmed by electronic structure calculations (Alavi and Ripmeester, 2007). To improve the storage capability of H_2_ in hydrates, one issue concerns the possibility of modifying the water cage relaxation. The dynamic properties of various hydrates have been investigated and water molecules reorients on a millisecond timescale (Sloan and Koh, 2008). Recently, it has been shown that water molecules relax on a nanosecond timescale in strong acid clathrate hydrates (Desmedt et al., 2004, 2013; Bedouret et al., 2014). Moreover, THF clathrate hydrate may be prepared by co-including strong acid molecules (Desmedt et al., 2015). Such chemical modification has an impact onto the lattice dynamics of the cages and on the melting points of the hydrates (Desmedt et al., 2015). However, to the best of our knowledge, no studies have been performed until now to investigate the H_2_ storage in such THF clathrate hydrates co-including strong acid species.

The description of the H_2_ insertion mechanism outlines the key role played by dynamical processes met in clathrate hydrates (Desmedt et al., 2012, 2017). At atmospheric pressure, only intra-cage diffusion of H_2_ is experimentally observed in the THF-H_2_ hydrate stability region (i.e., below *ca*. 270 K) (Pefoute et al., 2012). At higher pressure (typically several tens of bars), studies of molecular hydrogen diffusion into THF hydrates have been performed by means of NMR method (Okuchi et al., 2007), *in situ* neutron diffraction (Mulder et al., 2008), volumetric measurements (Nagai et al., 2008), theoretical calculations (Alavi and Ripmeester, 2007) and molecular dynamics simulations (Cao et al., 2013). These measurements, performed in various P-T thermodynamics conditions, lead to diffusion coefficient ranging from *ca*. 10^−6^ cm^2^/s to *ca*. 10^−12^ cm^2^/s. These results outline the importance of using an experimental method allowing the direct investigation of the spatial and time characteristics of the H_2_ diffusion within the hydrate. In this issue, confocal Raman microspectroscopy is a non-destructive approach and is particularly adapted for *in situ* investigation of transport process in nanoporous systems (Marti-Rujas et al., 2004, 2006, 2007). This vibrational technique is an interesting label-free tool to access the molecular composition, the molecular selectivity, the structural and dynamical information in gas hydrates (Chazallon et al., 2017; Petuya et al., 2017, 2018a,b; Petuya and Desmedt, 2019). Raman spectroscopy has been performed to study the hydrogen storage in hydrates (Florusse et al., 2004; Ogata et al., 2008; Strobel et al., 2009; Grim et al., 2012) and it has been shown that THF hydrates may act as a molecular sieving for hydrogen-containing gas mixtures (Zhong et al., 2017). This paper aims at studying the H_2_ diffusion mechanism in the THF clathrate hydrate by *in situ* confocal Raman microspectroscopy and imaging, and at investigating the impact of strong acid onto this diffusive process at 200 bar pressure. To address this issue, we use two different sII hydrogen hydrate formed with THF promoter: THF-H_2_ hydrate (non-acidic) and the THF-HClO_4_-H_2_ hydrate (acidic).

## Experimental Details

### Samples

Two solutions were prepared with the following molar ratio 8THF·136H_2_O (melting at 277 K) and 7THF^−^1HClO_4_·136H_2_O (melting at 271 K) using ultra-pure water (Milli-Q quality) and commercially available chemicals (70% HClO_4_ aqueous solution and 99.9% THF from Sigma-Aldrich). They were transferred under inert atmosphere in the lab-made high-pressure optical cell used for the Raman spectroscopic analysis. According to a procedure previously published (Desmedt et al., 2015), the hydrates have been formed under stirring conditions by cooling the sample temperature to 270 K and maintaining this temperature for 24 h with the help of a modified cryogenic stage (Linkam Scientific Instruments Ltd., UK). Once the hydrate is formed, hydrogen gas (99.9999% Air Liquide) was then applied at constant pressure (200 bar) with a PM High Pressure pump (Top Industrie, Vaux-le-Penil, France) which contains 100 cm^3^ of gas.

### Raman Scattering and Imaging

**Figure 1:**
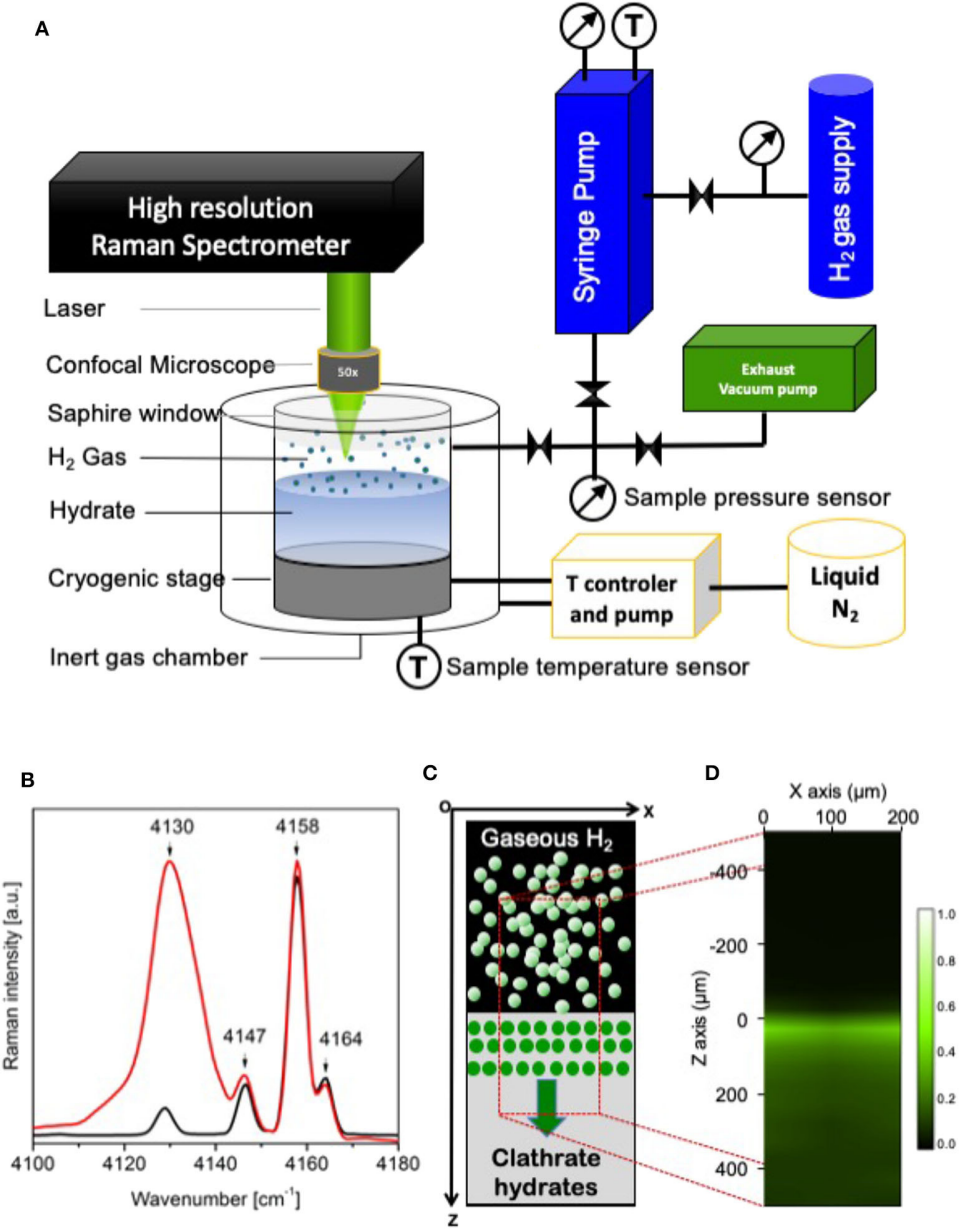
**(A)** Schematic representation of the experimental set-up used to monitor the insertion of molecular hydrogen within the THF hydrate. **(B)** Raman spectra of H_2_ gaseous (black, **A**) and of confined H_2_ in THF hydrate (red, **A**) at 270 K and 200 bar; **(C)** Schematic illustration of experimental measurement. The microraman imaged region is shown with red dashed lines; **(D)** Spectral image constructed using the integrated intensities of Raman bands at 4,130 cm^−1^ (encapsulated H_2_). Green color indicates inserted H_2_ in the hydrate and black color indicates no H_2_ inserted.

Raman data were collected with a Labram microspectrometer (Horiba Jobin Yvon, Villeneuve d'Ascq, France) and using a 514 nm laser source (10 mW power at the sample). A 50X objective (NA = 0.45, Olympus) permitted to focus the incident laser beam and to collect the Raman scattering. The Raman scattering was dispersed with a holographic grating of 1,800 g/mm and analyzed with a Peltier-cooled CCD detector (Andor, Belfast, UK), which permitted to measure the Raman spectra with a spectral resolution of 1 cm^−1^. The data was collected on a spectral range from 2,700 to 4,300 cm^−1^ to monitor the hydrogen stretching modes. To probe the H_2_ diffusion into the preformed hydrate as a function of time, micro-Raman spectra and imaging were collected *in situ* under the 200 bar H_2_ pressure at 270 K in the hydrate stability zone (Hashimoto et al., 2007). The measurement has been started simultaneously with the application of H_2_ pressure (corresponding to time *t* = 0 h in the following). For recording the spectral images, a motorized stage was used to map the sample in a point-by-point mode using a 20 μm step size in the two XZ plane perpendicular to the gas-hydrate interface contained in the XY plane (Figure 1). For each sample, an area of about 200 × 1,200 μm^2^ from the H_2_/hydrate interface to 1,000 μm depths in the hydrate by Raman micro-imaging. In the following, *Z* = 0 μm corresponds to the gas-hydrate interface. Raman intensity depth profiles of inserted H_2_ were corrected from the refraction by using standard procedure (Everall, 2000; Desmedt et al., 2007) with a refraction index *n* = 1,33 (Sloan and Koh, 2008) for the hydrate.

## Results and Discussion

**Figure 2:**
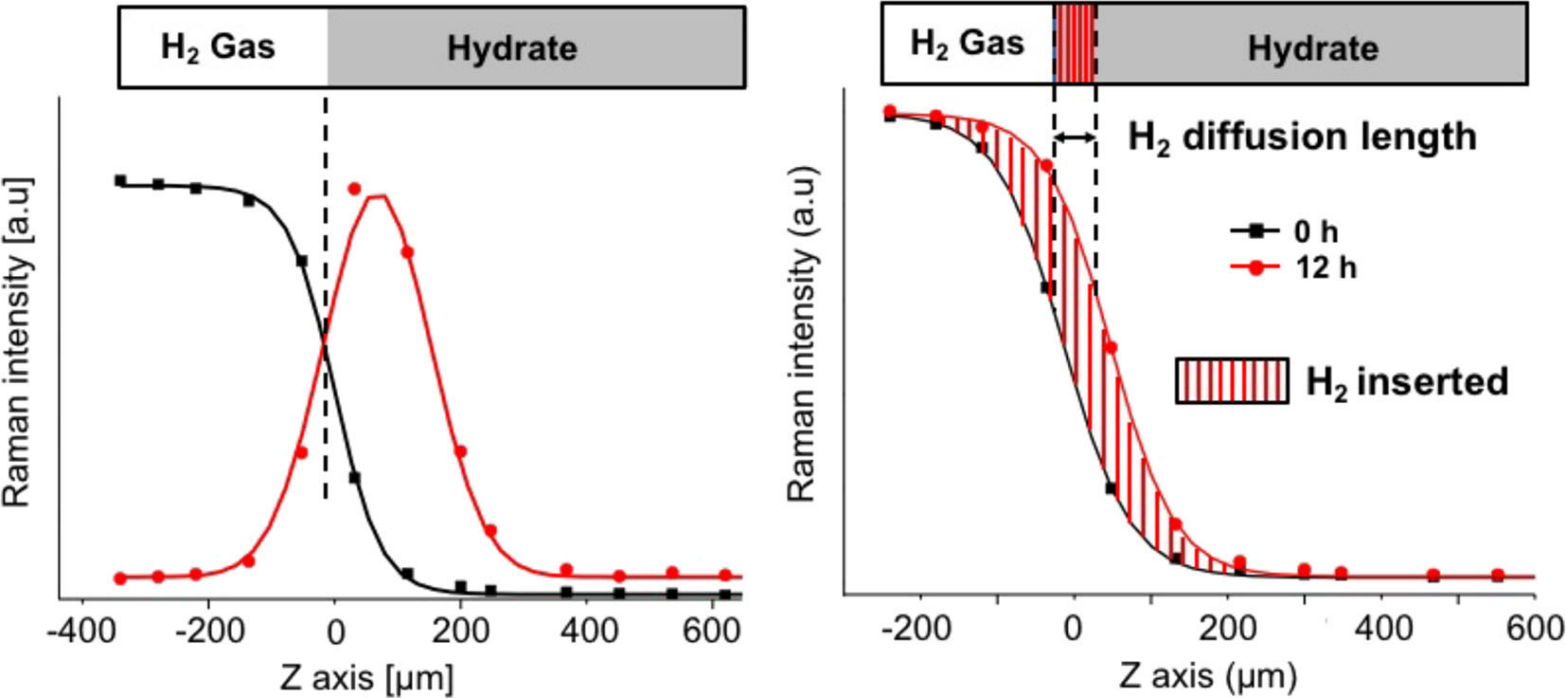
**Left**: H_2_ (black) and OH (red) Raman integrated intensity profiles (normalized) of the THF-HClO_4_-H_2_ hydrate at t = 0 h, 200 bar and 270 K. Negative Z values corresponds at the gaseous phase within the high-pressure optical cell, while positive Z values corresponds to the hydrate phase. **Right**: H_2_ Raman intensity profiles of the THF-HClO_4_-H_2_ hydrate at 0 h (black) and after 12 h (red) of 200 bar H_2_ pressure.

**Figure 3:**
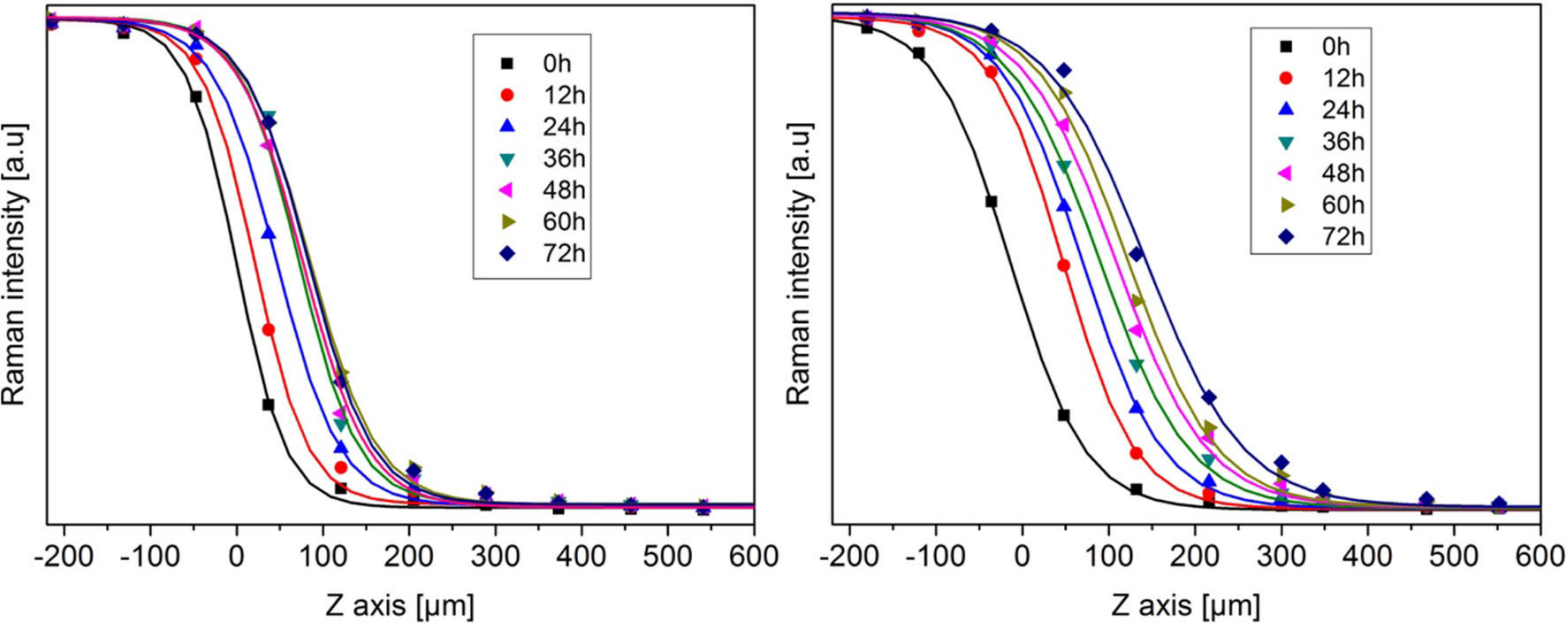
Time evolution of the H_2_ Raman intensity profiles for THF-H_2_ **(left)** and THF-HClO_4_-H_2_ **(right)** hydrates obtained at 270 K and 200 bar. The continuous lines are the sigmoidal functions fitted on the experimental points (see text for details).

**Figure 4:**
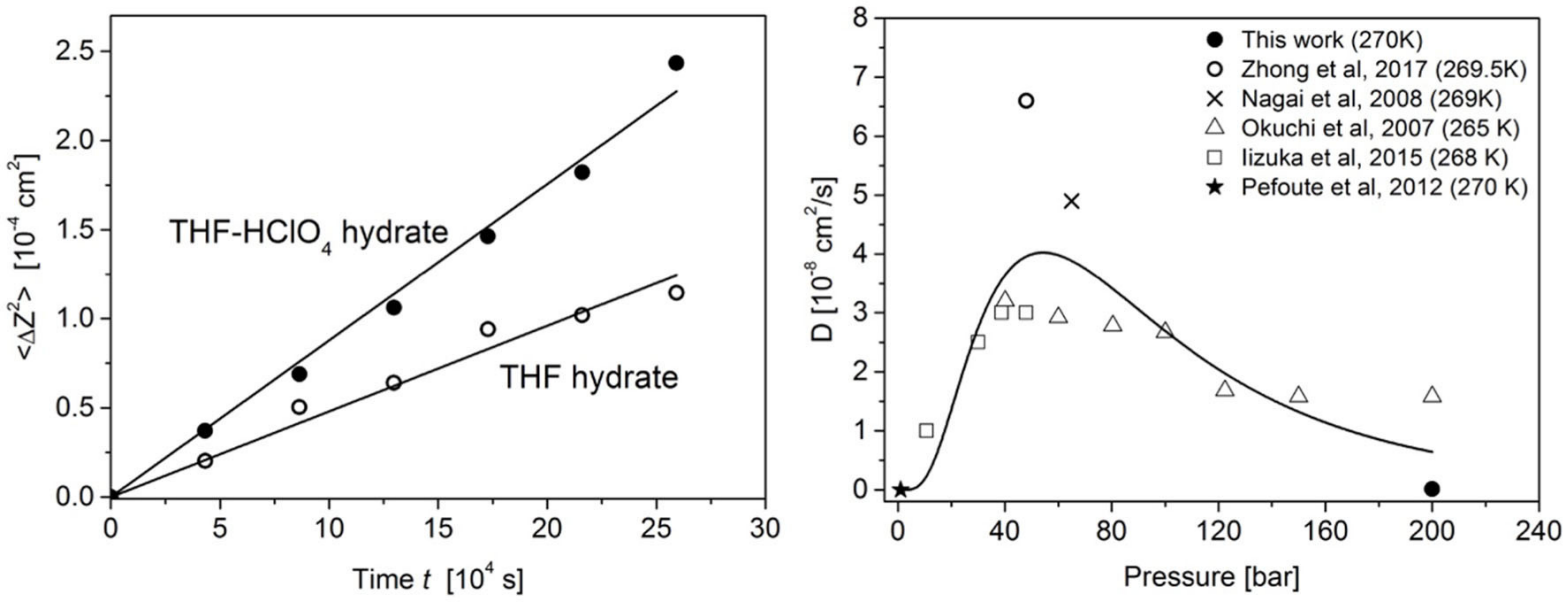
**Left**: H_2_ diffusion length of the THF-HClO_4_-H_2_ (filled symbols) and THF-H_2_ (open symbols) hydrates measured at 270 K and 200 bar. The lines represent the fitted Fick model (see text for details). **Right**: Pressure dependence of the H_2_ diffusion coefficient integrating data from various experimental investigations in the 265–270 K region (Okuchi et al., 2007; Nagai et al., 2008; Pefoute et al., 2012; Iizuka et al., 2015; Zhong et al., 2017). The line represents a guide-to-the-eyes showing that the diffusion coefficient goes through a maximum at *ca*. 55 bar.

In the present investigation, two hydrates have been considered: the THF hydrate (formed with a THF·17H_2_O solution) and the mixed THF-HClO_4_ hydrate (formed with a 0.875THF-0.125HClO_4_·17H_2_O solution). In these sII hydrates, the SCs are empty to welcome H_2_ molecules, the THF molecules occupy the LC and the acidic additive HClO_4_ is inserted within the LC by replacing one THF per unit cell in average (Desmedt et al., 2015). The insertion of molecular hydrogen within these two preformed hydrates has been probed thanks to real-time Raman imaging. An example of the spectral region corresponding to the gaseous H_2_ and confined H_2_ signatures is shown in Figure 1A for the THF-H_2_ hydrate formed within the lab-made high-pressure optical cell after 1 day at 200 bars and 270 K. The rotation-vibration coupled bands of gaseous H_2_ are observed at 4,130, 4,147, 4,155, and 4,164 cm^−1^. In the case of the THF-H_2_ hydrate, the band at 4,130 cm^−1^ is assigned to the Raman signal of H_2_ confined in the sII hydrate SCs (Ogata et al., 2008; Strobel et al., 2009; Grim et al., 2012). To monitor the insertion of hydrogen within the hydrate, Raman imaging has been performed in the plane perpendicular to the gas/hydrate interface (see Figure 1B). An example of visualization of the H_2_ molecules confined in the hydrate is shown in Figure 1C through the Raman mapping of the ratio of the band intensity at 4,130 cm^−1^ and of the one at 4,155 cm^−1^. The Raman intensity being proportional to the encapsulated species concentration (Zhong et al., 2017), such a Raman image is a direct signature of the spatial H_2_ distribution within the preformed THF hydrate; after 1 day of pressurization, one can observe that H_2_ has mainly be inserted within few tens of micrometers below the hydrate surface. In order to spatially analyse the extension of H_2_ insertion, one needs to locate the gas/hydrate interface at a micrometric scale. In this purpose, the intensity profiles of the H_2_ vibron and of the OH stretching modes have been measured with the help of the projection of the acquired Raman spectra in the XZ plane along the Z axis (see Figure 1B). An example of such averaged profiles is shown in Figure 2 (left) for the measurement at initial stage (*t* = 0 h) of H_2_ pressurization at 200 bar and at T = 270 K. A pseudo-Voigt function has been used to reproduce the experimental data of the OH integrated intensity profile and the H_2_ intensity profile has been fitted with a sigmoidal function. The fitted functions reproduce with a good agreement the experimental data (the residual error between experimental and modeled curves reached a value of 10^−3^). The gas/hydrate interface is then clearly identified at the intersection of these two curves: it corresponds to the Z coordinate of the inflection point of the H_2_ sigmoidal curve. This procedure allows to accurately determine the Z position of the gas/hydrate interface and is set as the reference distance *Z* = 0 μm. Moreover, the H_2_ vibron intensity profile can be used as a signature of the H_2_ diffusion front position as a function of time. This is shown in Figure 2 (right): the H_2_ intensity profiles of THF-HClO_4_-H_2_ hydrate after 12 h of pressurization is clearly *Z*-shifted with respect to the profile at initial time *t* = 0 h. This *Z* difference of the H_2_ diffusion front is the signature of the insertion of H_2_ molecule within the hydrate sample; it is defined as the mean H_2_ diffusion length, Δ*Z*(*t*):

(1) $$\begin{array}{r} \langle \Delta Z\left(t\right)\rangle =Z\left(t\right)-Z\left(0\right)\; \end{array}$$

where *Z*(*t*) corresponds to the inflection point of the H_2_ sigmoidal curve at time *t*. To subsequently analyse the time-evolution of the H_2_ insertion, such H_2_ Raman intensity profiles have been recorded every 12 h over a period of 3 days. In order to evaluate the impact of the strong acid HClO_4_ additive, these measurements have been performed onto the preformed THF hydrate and onto the preformed THF-HClO_4_ hydrate, both pressurized with H_2_ gas at 270 K and 200 bar. The time evolution of the H_2_ intensity profiles is shown in Figure 3 for the THF-HClO_4_-H_2_ hydrate and for the THF-H_2_ hydrate. It can be observed a marked difference between these time evolutions: the H_2_ diffusion front extend over a wider range in the case of the THF-HClO_4_ hydrates compared to the case of the THF hydrate, i.e., without acidic additives. In order to quantitatively analyse the time evolution of the H_2_ diffusion front within the hydrates, each intensity profile has been fitted by using the sigmoidal function as previous described, allowing to measure the position of the H_2_ diffusion front as a function of time. Such procedure allows to measure the H_2_ diffusion lengths (Equation 1) as a function of time. Their mean-squared values 〈Δ*Z*(*t*)^2^〉 are reported in Figure 4. These curves show clearly the improvement of the H_2_ insertion when acidic additive are included in the THF hydrate: the values are higher for the THF-HClO_4_ hydrate than for the THF hydrate.

Furthermore, such curves provide the opportunity to investigate the H_2_ insertion mechanism. Such diffusive mechanism may include various elementary processes including the inter-cage diffusion and possible contributions associated with grain boundaries, chemical and structural defects. Let first consider two principal diffusive models by analogy with nanoporous systems (Marti-Rujas et al., 2007): conventional diffusion or single-file-like diffusion. The conventional diffusion corresponds to the Fick law for which the mean-squared H_2_ diffusion length is given by the Einstein diffusion model in a three-dimensional system:

(2) $$\begin{array}{r} \langle {\Delta Z\left(t\right)}^{2}\rangle =6Dt\; \end{array}$$

where *D* is the H_2_ diffusion coefficient. The Fick behavior may reproduce not only the inter-cage diffusion, but also the diffusion associated with grain boundaries and structural defects. The single-file-like diffusive model may be considered for the H_2_ inter-cage diffusion. Indeed, the H_2_ molecule dimension is comparable to the diameter of the polygonal faces of the water cages. In such a model, H_2_ molecule diffusion within a water cage may proceed under the condition that there is a vacant guest site in the neighboring cage—unlike in the case of Fick behavior for which H_2_ molecules can overtake each other in the cages. This phenomenon involves correlations between the H_2_ displacements within the hydrate and thus the time-dependence of 〈Δ*Z*(*t*)^2^〉 differs from the Fick diffusion:

(3) $$\begin{array}{r} \langle {\Delta Z\left(t\right)}^{2}\rangle =6Mt^{1/2}\; \end{array}$$

where *M* correspond to the H_2_ mobility in the three-dimensional hydrate system. The time dependence of 〈Δ*Z*(*t*)^2^〉 exhibit a clear linear behavior (it has not been possible to reproduce the experimental data with the single-file-like model). It thus implies that H_2_ molecules can overtake each other in a cage and mainly follows a fick behavior. such a diffusive behavior is in full agreement with NMR (Okuchi et al., 2007), *in situ* neutron diffraction (Mulder et al., 2008), volumetric measurements (Nagai et al., 2008), Raman (Zhong et al., 2017), and molecular dynamics (Cao et al., 2013). The Fick law given by Equation (2) have been fitted to the experimental data with an excellent agreement (see Figure 4-Left). The fitted diffusion coefficients are of 7.98 ± 0.03 10^−11^ cm^2^/s for the THF hydrate and 1.46 ± 0.03 10^−10^ cm^2^/s for the THF-HClO_4_ hydrate at 270 K and 200 bar. The obtained diffusion coefficient for the H_2_ insertion in the THF-H_2_ hydrate is within the broad range of values obtained by various experimental methods and reported in Figure 4-Right. The present study confirms the trend of an optimum pressure for promoting the hydrogen diffusion at *ca*. 55 bar. Such a behavior reflects a trend regarding the transport of hydrogen molecules between the cages: the inter-cage diffusion is slower when a large fraction of the small cage is already filled, or even doubly filled with H_2_ as reported from Molecular Dynamics simulations (Cao et al., 2013). Once the small cage of the sII THF hydrate are filled, structural or chemical defects and grain boundaries should play a key-role for insuring the Fickian H_2_ diffusion through and within the sII THF hydrate. Indeed, the behavior observed with the THF-HClO_4_ hydrate confirms the importance of such defects: a clear quantitative enhancement of the H_2_ diffusion in the THF hydrate is measured thanks to the co-inclusion of HClO_4_ in the sII structure (Figure 4-Left). As reported in previous studies (Desmedt et al., 2015), the inclusion of HClO_4_ into the THF hydrate lead to generate perchlorate anions confined in the LC and acidic protons delocalized within the water cage structure (this delocalization is at the origin of the super-protonic conductivity met in strong acid hydrates; Desmedt et al., 2004, 2013). In such a case, the cages are constituted not only of water molecules, but also of hydronium ions. The energy barrier related to H_2_ molecules diffusion through the water cage being higher for SC than for LC (Okuchi et al., 2007), the inclusion of such ionic defects in the hydrate lead to modify the flexibility of the cage, as reported from Raman measurements of the water cage phonons (Desmedt et al., 2015). This increased flexibility of the water cage—especially of the SCs welcoming the H_2_—may lead to decrease the energy barrier required for an H_2_ molecule to cross the faces of the water cage and thus to facilitate the inter-cage diffusion, as reflected by the enhanced diffusion coefficient measured in the THF-HClO_4_.

## Conclusion

The present investigation represents the first report to shed light on the impact of ionic defects onto the hydrogen insertion within hydrate. This study has been realized by comparing the H_2_ diffusion within the THF hydrate and the mixed THF-HClO_4_ hydrate, both being sII structure. Raman confocal microspectroscopy and imaging has been a powerful tool. It yields the measurement of the H_2_ Fickian diffusion coefficients within the two hydrates at 270 K and 200 bar: 7.98 ± 0.03 10^−11^ cm^2^/s for the THF hydrate and 1.46 ± 0.03 10^−10^ cm^2^/s for the THF-HClO_4_ hydrate. In the case of the THF hydrate, it is shown that the H_2_ diffusion within the hydrate is optimum for a pressure of *ca*. 55 bar by compiling this result with those from the literature. Moreover, this investigation clearly shows the enhancement of the H_2_ insertion within the THF hydrate thanks to the co-inclusion of acidic additive: it acts as a “flexibilizer” of the water cage through the addition of chemical water H-bond defects (Desmedt et al., 2015) promoting the H_2_ inter-cage diffusion. Such results are particularly exciting and promising for applications in storage of hydrogen and open new routes for developing efficient hydrate-based hydrogen storage materials with new type of promoter.

## Data Availability Statement

The original contributions presented in the study are included in the article/supplementary material, further inquiries can be directed to the corresponding author/s.

## Author Contributions

TN and CP have performed the sample preparation, the Raman scattering experiments, and data acquisition. DT has contributed to the Raman spectrometer configuration and data management. TN and AD has analyzed, interpreted the data, and has written the manuscript. All authors contributed to the article and approved the submitted version.

## Conflict of Interest

The authors declare that the research was conducted in the absence of any commercial or financial relationships that could be construed as a potential conflict of interest.
